# Protein kinase C-β distinctly regulates blood-brain barrier-forming capacity of Brain Microvascular endothelial cells and outgrowth endothelial cells

**DOI:** 10.1007/s11011-022-01041-1

**Published:** 2022-06-28

**Authors:** Rais Reskiawan A. Kadir, Mansour Alwjwaj, Ulvi Bayraktutan

**Affiliations:** 1 Academic Unit of Mental Health and Clinical Neuroscience, The University of Nottingham, Nottingham, UK; 2 Academic Unit of Mental Health and Clinical Neuroscience, Clinical Sciences Building, School of Medicine, The University of Nottingham, Hucknall Road, NG5 1PB Nottingham, UK

**Keywords:** Endothelial progenitor cells, Blood-brain barrier, Protein kinase C, Oxidative stress, Stroke, Cell-based therapy

## Abstract

Outgrowth endothelial cells (OECs) provide an endogenous repair mechanism and thus maintain endothelial barrier integrity. As inhibition of protein kinase C-β (PKC-β) activity has been shown to attenuate endothelial damage in various pathological conditions including hyperglycaemia and ischaemic injury, the present study comparatively assessed the effect of LY333531, a PKC-β inhibitor, on the cerebral barrier integrity formed by OECs or human brain microvascular endothelial cells (HBMECs). To this end, an in vitro model of human BBB established by co-culture of astrocytes and pericytes with either OECs or HBMECs was exposed to 4 h of oxygen-glucose deprivation with/out LY333531 (0.05 µM). The inhibition of PKC-β protected the integrity and function of the BBB formed by HBMECs, as evidenced by increases in transendothelial electrical resistance and decreases in sodium fluorescein flux. It also attenuated ischaemia-evoked actin cytoskeleton remodelling, oxidative stress, and apoptosis in HBMECs. In contrast, treatments with LY333531 exacerbated the deleterious effect of ischaemia on the integrity and function of BBB formed by OECs while augmenting the levels of oxidative stress, apoptosis, and cytoskeletal reorganisation in OECs. Interestingly, the magnitude of damage in all aforementioned parameters, notably oxidative stress, was lower with low dose of LY333531 (0.01 µM). It is therefore possible that the therapeutic concentration of LY333531 (0.05 µM) may neutralise the activity of NADPH oxidase and thus trigger a negative feedback mechanism which in turn exacerbate the detrimental effects of ischaemic injury. In conclusion, targeting PKC-β signalling pathway in ischaemic settings requires close attention while using OECs as cellular therapeutic.

## Introduction

Endothelial progenitor cells (EPCs) constitute a very scarce bone marrow-derived stem cell in the peripheral blood and may serve as potential therapeutics for stroke due to their unique ability to promote post-stroke recovery by both directly differentiating into cerebral endothelial cells, and releasing wide range of paracrine factors to further potentiate neuro-angiogenesis (Bayraktutan 2019; Alwjwaj et al. 2021). The limited availability of EPCs in peripheral blood, estimated to be 1 cell/30 ml blood, necessitate their ex vivo expansion to generate a population of homogenous cells. Attempts to generate EPCs ex vivo produce two morphologically and functionally distinct EPC subtypes, namely early EPCs (eEPCs) and late EPCs or outgrowth endothelial cells (OECs). While the former appears in culture conditions within 3–5 days of seeding blood-derived mononuclear cells (BMNCs) and possess spindle-shaped morphology, the latter appears between 14 and 28 days after seeding of BMNCs and display typical cobblestone morphology (Medina et al. 2010; Abdulkadir et al. 2020). Equipped with a remarkable proliferative and migratory potential and a capacity to form *de novo* vascular network, OECs are considered as the actual and functional subtypes of EPCs (Lin et al. 2014; Medina et al. 2017; Banno and Yoder 2018). Since OECs also serve as a continuous source of replenishment of the damaged cerebral endothelial cells, the most prominent cellular component of the blood-brain barrier (BBB), it is bioavailability may determine overall cerebrovascular function and neurological recovery following ischaemic stroke (Asahara et al. 1999; Sargento-Freitas et al. 2018; Kukumberg et al. 2020). In this regard, a better understanding of the molecular mechanism regulating the capacity of OECs to form a tight and functional BBB is of paramount importance in an effort to substantiate their barrier-forming and barrier-restorative capacity.

Accumulating evidence indicate that protein kinase C (PKC) plays a pivotal role in regulating the proliferation, migration, survivability, cytoskeletal structure, and tight junction formation of brain endothelial cells (Srivastava et al. 2013; Rakkar and Bayraktutan 2016). However, the substantial increases in PKC expression have also been observed in the infarct area of the brain of deceased ischemic stroke patients, implicating the involvement of this enzyme in major pathologies associated with cerebral ischaemic injury (Krupiński et al. 1998). The mechanism of PKC activation depends on their subtypes, in that Ca^2+^ and diacylglycerol (DAG) are required for the activation of classical isoform, while only DAG is required for novel isoform activation, and neither Ca^2+^ or DAG for atypical subtype (Newton 2018). Compared to all other isoforms, PKC-β subtype contributes the most to overall PKC activity in hyperglycaemic settings and plays a crucial role in exacerbation of the in vitro cerebral barrier damage by regulating the expression of NADPH oxidase activity, the main source of reactive oxygen species (ROS) in brain vasculature (Shao and Bayraktutan 2013, 2014). Remarkable increases in total PKC activity have also been documented in brain endothelial cells subjected to ischaemic injury where increases in PKC-β appear to be the main contributor (Fleegal et al. 2005).

In light of the abovementioned widespread association of PKC-β to vascular pathologies and such seminal roles of functional EPCs in the detection and replacement of dead or damaged cerebral endothelial cells, this study explored the specific impact of this particular PKC isoform on cerebral barrier-forming capacity of human brain microvascular endothelial cells (HBMECs, resident ECs) or OECs during ischaemic injury. To provide a mechanistic understanding for the putative beneficial effects that may be generated by PKC-β inhibition in ischaemic settings, the state of cytoskeletal organisation and the levels of total antioxidant capacity, pro-oxidant NADPH oxidase activity, superoxide anion level, cellular viability, and apoptosis have also been explored.

## Materials and methods

### Cell culture

HBMECs, human astrocytes (HAs), and human pericytes (HPs) were purchased from TCS CellWorks Ltd. (Buckingham, UK) and were cultured in their respective specialised media (Sciencell Research Laboratories, San Diego, USA) in a humidified atmosphere (75% N_2_, 20% O_2_, 5% CO_2_) at 37 °C. OECs were isolated from human peripheral blood-derived mononuclear cells and were cultured on fibronectin-coated tissue culture plates as before (Abdulkadir et al. 2020). OECs between passages 6 and 9 were used in the current study.

To simulate ischaemic stroke injury, cells were exposed to 4 h OGD (94.95% N_2_, 0.05% O_2_, 5% CO_2_) in the presence or absence of a PKC-β inhibitor, LY333531 (0.05 µM). This particular concentration was selected as therapeutic dose due to the observation of an effective inhibition of oxidative stress and apoptosis in HBMECs as well as an adequate protection of cerebral barrier integrity against either hyperglycaemic or ischaemic/reperfusion injury from our previous studies (Shao and Bayraktutan 2013, 2014; Mathur and Bayraktutan 2016). The unexpected detrimental effects of this therapeutic concentration in OECs may suggest a complete neutralisation of NADPH oxidase activity and consequent activation of a negative feedback mechanism that leads to exacerbation of ischaemia-mediated deleterious effects in OECs. We therefore utilised the dose 0.01 µM in further experiments to scrutinise this concept. OGD experiments were performed in a custom-made multigas incubator (MCO-18 M, Sanyo UK) and using the D-glucose free medium (RPMI 1640, Invitrogen).

**Establishment of an*in vitro*model of BBB**.

An in vitro model of human BBB composed of HAs, HPs, and HBMECs or OECs was established as before (Shao and Bayraktutan 2013). In brief, about 7.5 × 10^4^ HAs were seeded on the basal side of polyester Transwell inserts (0.4 μm pore size, 12 mm diameter polyester membrane, High Wycombe, UK). The following day, the inserts were inverted to return to their original orientation and were placed into a 12-well plate containing fresh medium to grow to about 90% confluence. HBMEC or OECs (~ 5 × 10^4^ cells) were subsequently seeded on the apical part of the insert, and both cell layers were left to grow to full confluence. To set up the triple-culture model, these Transwell inserts were transferred to 12-well plates containing confluent pericytes. To reveal the precise nature of correlation between PKC-β and HBMECs or OECs, we have avoided using the mixture of HBMECs and OECs to set up the in vitro model of human BBB.

### Assessment of BBB integrity and function

The integrity and function of the BBB were assessed as before by measurements of transendothelial electrical resistance (TEER, World Precision Instruments, Hertfordshire, UK) and paracellular flux of low molecular weight permeability marker, sodium fluorescein (NaF, 376Da), respectively (Abdulkadir et al. 2020).

### Immunocytochemistry

The organisation of F-actin filaments was studied as an important marker of cytoskeletal organisation. For this, the cells were grown to about 80% confluence on glass coverslips before subjecting to normal and experimental conditions. They were then successively fixed and permeabilised in 4% paraformaldehyde/PBS and 0.1% Triton X-100/PBS for 20 min before staining with 1× rhodamine phalloidin for 60 min (Abcam, Cambridge, UK). The coverslips were mounted on glass slides and visualised with fluorescence microscopy (Zeiss Axio Observer, Carl Zeiss Ltd, Cambridge, UK). The number of stress fibres was counted manually and normalised to control.

2’-[4-ethoxyphenyl]-5-[4-methyl-1-piperazinyl]-2,5’-bi-1 H-benzimidazole trihydrochloride trihydrate (Hoechst 33,258, Sigma) staining was used to visualise the fragmented DNA, a hallmark of apoptosis (Binti Kamaruddin et al. 2020). Identical number of cells was seeded on coverslips and grown to ~ 90% confluence before exposing to experimental conditions. The cells were then fixed in 4% paraformaldehyde for 20 min and were incubated with 10 µg/mL of Hoechst 33,258 for 5 min in room temperature. The coverslips were then mounted on glass slides and observed under fluorescence microscopy (Zeiss Axio Observer, Carl Zeiss Ltd, Cambridge, UK).

To study the endothelial characteristic of the cells, the 95% confluent of HBMECs or OECs were incubated with 1,1′-dioctadecyl-3,3,3′,3′-tetramethylindo-carbocyanine-labelled- acetylated-low density lipoprotein (Dil-Ac-LDL, 1 mg/mL, Invitrogen, Loughborough, UK) for 4 h before staining with FITC-conjugated Ulex europaeus agglutinin (FITC-UEA-1, 1 mg/mL, Sigma). The cells were subsequently fixed with 4% formaldehyde and mounted on glass slides using a mounting medium (Vector Laboratories, Peterborough, UK). ImageJ software (version 1.52k, NIH, Maryland, USA) was used to quantify the florescence signal. The data, presented as corrected total cell fluorescence (CTCF), were calculated using the following formula *CTCF = A- (B X C)* where A, B and C successively represent integrated density, area of seleceted cell, and mean of fluorescence of background readings. The data were subsequently normalised by the number of cells displayed (Migneault et al. 2020).

### Tube formation assay

150 µL of growth factor-reduced Matrigel (BD Biosciences) was added to 48-well plates and incubated for 90 min at 37 °C. 9 × 10^4^ of HBMECs or OECs were then seeded in the plates and incubated for 8 h. Tubule networks were visualised by a light microscope. The total number and length of tubule networks, defined by sum of number or length of segments, isolated elements and branches detected in the analysed area, were assessed using ImageJ software (version 1.52k, NIH, Maryland, USA) (Chevalier et al. 2014).

### Detection of NADPH oxidase activity and superoxide anion level

NADPH oxidase activity was measured using the lucigenin chemiluminescence assay as previously described (Kadir et al. 2021). The HBMEC or OEC homogenates (50 µg) were incubated at 37 °C in assay buffer containing lucigenin (200 µM, Sigma), potassium phosphate buffer (300 mM, pH 7.0, Sigma), sucrose (1 M, Sigma), and ethylene glycol tetraacetic acid (50 mM, Sigma). The specific inhibitors for nitric oxide synthase (NG-nitro-L-arginine methyl ester, 10 mM, Sigma), xanthine oxidase (allopurinol, 10 mM, Sigma), mitochondrial respiratory chain complex 1 (rotenone, 10 mM, Sigma), and cyclooxygenase (indomethacin, 10 mM, Sigma) were also added to assay buffer to diminish the contributions of other ROS-generating enzymes to overall superoxide anion generation. NADPH (100 µmol/L, Sigma) was injected to the well plate to initiate the reaction after 15 min. The reaction was subsequently monitored for 2 h, and the rate of reaction was calculated using a luminometer (FLUOstar Omega, BMG Labtech, Aylesbury, UK).

To determine the level of superoxide anion, cytochrome-*C* reduction assay was employed (Rakkar and Bayraktutan 2016). In brief, equal amounts of homogenate (100 µg) were incubated with assay buffer containing HEPES (1 M, Calbiochem, UK), ethylene glycol tetraacetic acid (50 mM, Sigma), sucrose (1 M, Sigma), mannitol (1 M, Sigma), and cytochrome *C* (800 µM, Sigma), for 1 h at 37 °C. Superoxide anion generation was measured as the reduction of cytochrome *C* and monitored with the change in absorbance at 550 nm using a plate reader (FLUOstar Omega, BMG Labtech, Aylesbury, UK).

### Measurement of total antioxidant capacity

The total anti-oxidant capacity was measured in HBMECs and OECs using a total antioxidant capacity assay kit (Abcam, Cambridge, UK) as per manufacturer’s instructions. In brief, cells grown under normal and experimental conditions were pelleted by trypsinisation which were then re-suspended with double distilled water (ddH_2_O) before centrifugation for 4 min at 10,000 rpm to obtain the supernatant. 100 µL of working solution (the mixture of Cu^2+^ reagent and assay diluent) was then mixed with samples and standards and incubated at room temperature for 90 min before measuring total anti-oxidant capacity in a plate reader (FLUOstar Omega, BMG Labtech, Aylesbury, UK) at 570 nm.

### Caspase 3/7 assay

Apo-ONE homogeneous caspase-3/7 assay kit was used to measure the caspase-3/7 activities (Promega, Southampton, UK). Briefly, cells were grown to 95% confluence in 96-well black opaque plates and subjected to experimental conditions. The media was subsequently replaced with 100 µL of caspase 3/7 assay buffer containing the non-fluorescent caspase substrate, rhodamine 110, bis-(N-CBZL-aspartyl- L-glutamyl-L-valyl-L-aspartic acid amide; Z-DEVD-R110) to initiate the reaction. Plates were immediately frozen at -80 °C overnight, and were completely thawed on a plate shaker in the following day. The fluorescence produced by the caspase-3/7-mediated cleavage of the non-fluorescent caspase substrate to fluorescent rhodamine-110 was read in a FLUOstar Omega plate reader (BMG Labtech Ltd., UK) with excitation/emission: 485/520 nm. Blanks were subtracted from the readings before normalising activity against “mg protein”. Since the assay kit was found to be sensitive to the media employed (ECM and RPMI media) (Abdullah et al. 2015; Rakkar and Bayraktutan 2016), the RPMI media used as the control medium on the current study.

### Cell viability

The viability of cells was measured using Calcein AM assay (Calbiochem). In brief, ~ 95% confluent cells were seeded in 96-black opaque well-plate and subjected to experimental conditions. The media was replaced with 10 µg/mL of Calcein AM, and plates were immediately frozen at -80^o^C. In the following day, the plates were thawed in the plate shaker and fluorescence was measured using a FLUOstar Omega plate reader (BMG Labtech Ltd., UK) at 485/530 nm excitation/emission. Blanks were subtracted from the readings.

### Statistical analyses

Data are displayed as mean ± SEM from a minimum of three independent experiments. Statistical analyses were performed by one-way analysis of variance (ANOVA) followed by Tukey post hoc analysis using the GraphPad Prism 8.0 statistical software package (GraphPad Software Inc., La Jolla, Ca, USA). P < 0.05 was considered statistically significant.

## Results

### OECs possess endothelial cell characteristics

**Fig. 1:**
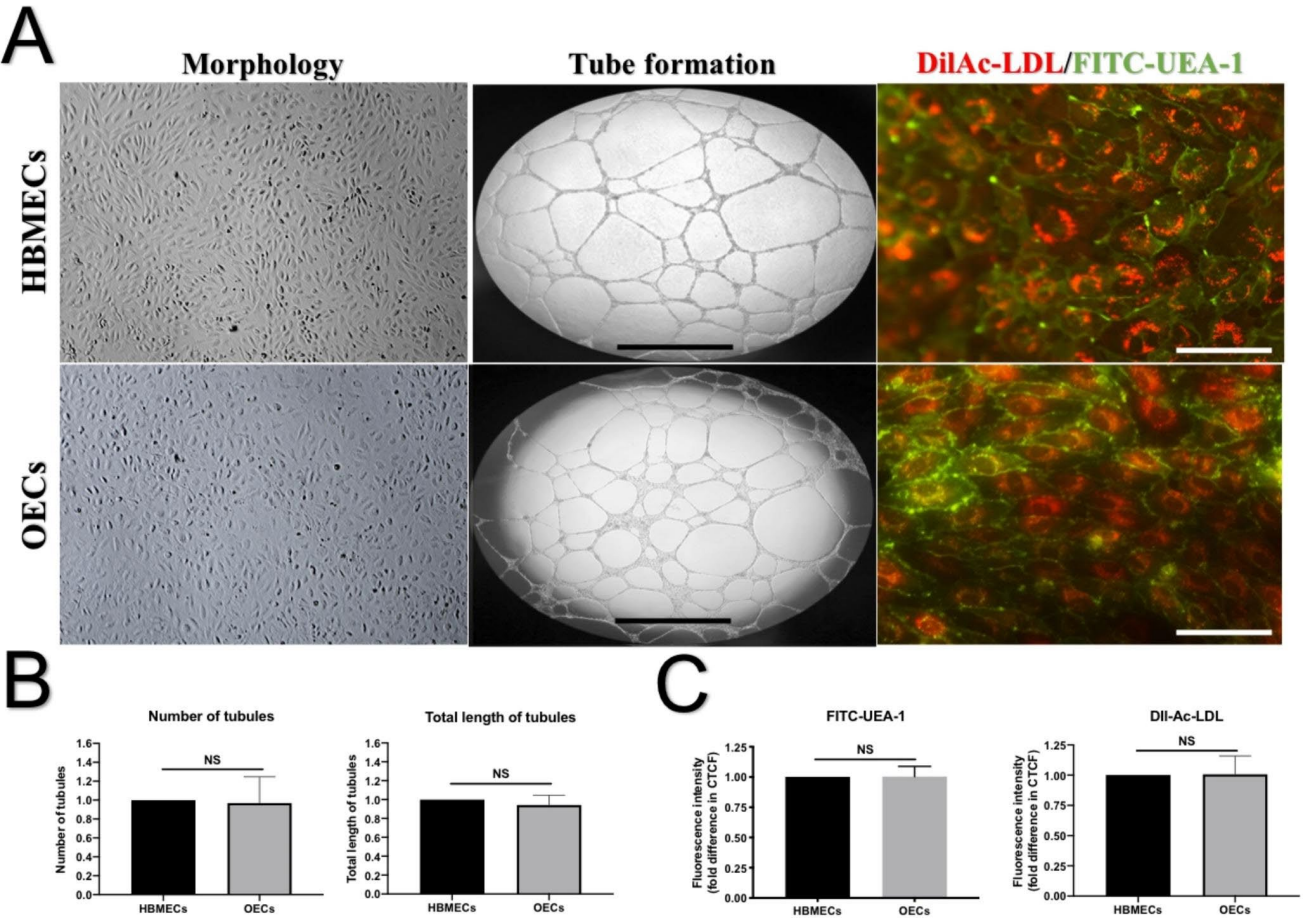
Representative images for HBMEC and OEC morphology, tube formation, and endothelial characterisation. (A) Both HBMECs and OECs show a cobblestone morphology, well-established tubules structure on matrigel, and positive staining with FITC-conjugated ulex europaeus agglutinin-1 (FITC-UEA-1) and Dil-labelled acetylated-low density lipoprotein (Dil-Ac-LDL). Quantitative analyses of the number and length of tubule networks (B) as well as the fluorescence intensity of FITC-UEA-1 and Dil-Ac-LDL signal (C) show similar readings for both cells. Scale bar 100 μm. NS: not significant

To probe the characteritsics of the OEC population, we stained the cells with Dil-Ac-LDL and FITC-UEA-1 and assessed their tubules network on Matrigel. Similar to HBMECs, OECs displayed typical cobblestone morphology, formed tubules, uptook Dil-Ac-LDL, and stained positive for FITC-UEA-1, suggesting that OECs possess true endothelial characteristics (Fig. 1). We and others have previously shown that OECs isolated in this fashion also express specific markers for immaturity (CD133), stemness (CD34), and endothelial maturity (CD31), but not for hematopoietic cells (CD45) (Medina et al. 2010; Abdulkadir et al. 2020).

**PKC-β inhibition maintains BBB integrity formed by HBMECs, but aggravates the detrimental role of OGD in BBB established by OECs**.

**Fig. 2:**
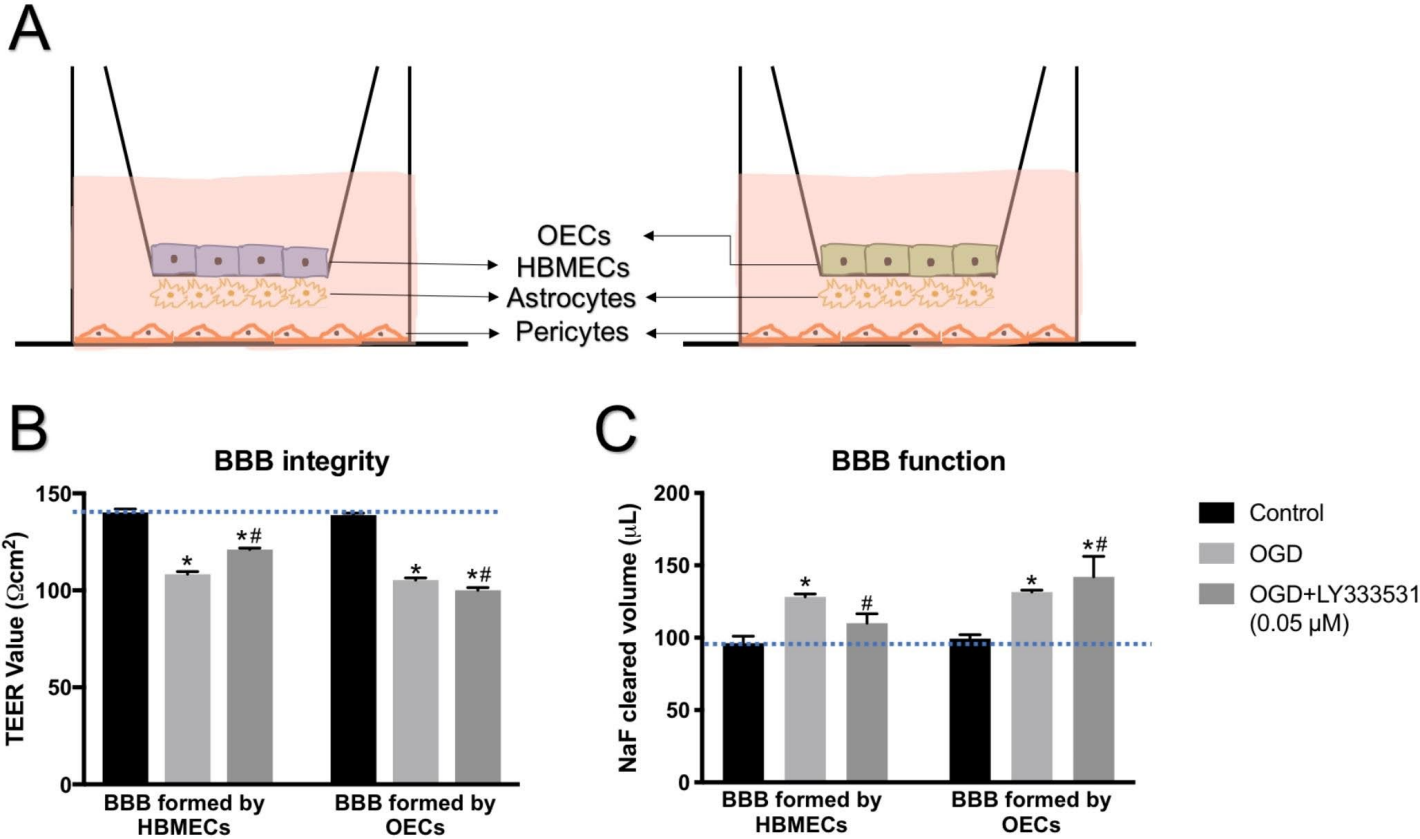
The schematic diagram of an in vitro model of human BBB consisted of HBMECs or OECs in the presence of astrocytes and pericytes (A). The inhibition of PKC-β (LY333531, 0.05 µM) protected BBB integrity (B) and function (C), as observed by the increases in TEER value and decreases in the flux of permeability marker, sodium fluorescein. Although OECs form equally tight and functional BBB (as indicated by the blue line), the inhibition of PKC-β (LY333531, 0.05 µM) exacerbates ischaemia-evoked damage in BBB established with OECs. *p < 0.05 versus control, #p < 0.05 versus OGD

Although LY333531 is recognised as a protective agent to the BBB integrity formed by HBMECs against various pathological condition such as ischaemic and hyperglycaemic injury, the specific effect of this compound on the barrier-forming capacity of OECs remains unknown. We therefore comparatively assessed the impact of PKC-β inhibition on the BBB established either by HBMECs or OECs. The specific inhibition of PKC-β protected the integrity and function of a triple cell-culture model of human BBB composed of HBMECs, astrocytes, and pericytes against OGD injury, as observed by significant increases in TEER value and concomitant decreases in sodium fluorescein flux, respectively. Although replacement of HBMECs in this model with OECs lead to formation of equally functional BBB, as evidenced by similar readings in TEER value and sodium fluorescent flux (indicated by blue line), inhibition of PKC-β with LY333531 worsened the barrier-disruptive effect of OGD (Fig. 2).

**PKC-β inhibition protects HBMECs cytoskeleton structure but exacerbates the deleterious effect of OGD on OECs architecture**.

**Fig. 3:**
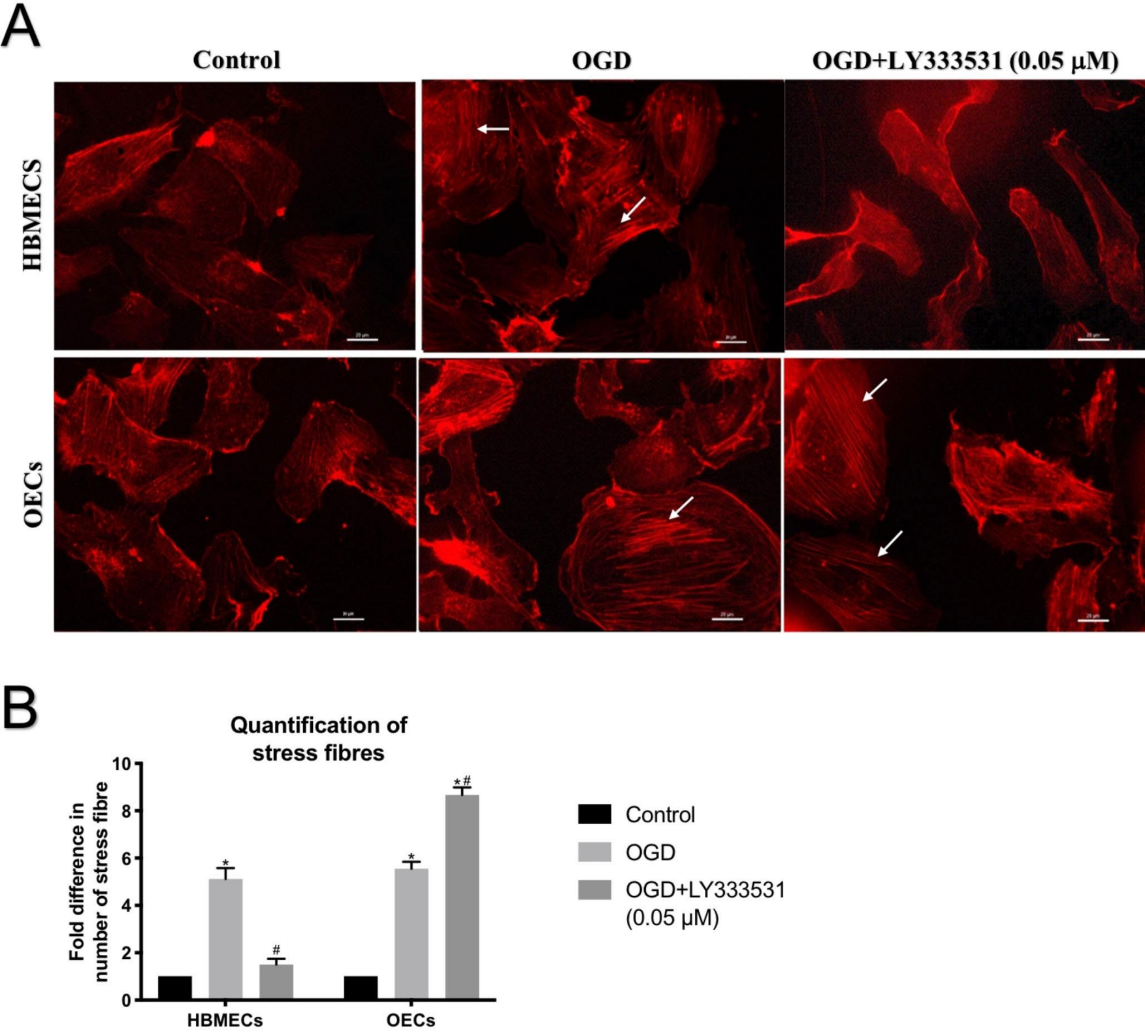
Representative images for actin cytoskeleton in HBMECs and OECs subjected to ischaemic injury in the presence or absence of LY333531. (A) While the presence of LY333531 (0.05 µM) diminished actin stress fibre formation evoked by OGD in HBMECs as indicated by white arrow, it produced the opposite effects in OECs. (B) The number of stress fibres formation in both cells. *p < 0.05 versus control, #p < 0.05 versus OGD. Scale bar: 20 μm

Since the formation of actin filaments is important for the maintenance of BBB integrity, we further investigated the effect of PKC-β inhibition on the actin cytoskeleton organisation of HBMECs and OECs. The exposure of HBMECs and OECs to OGD injury evoked dramatic changes on actin cytoskeleton organisation and promoted stress fibres formation in both cells. While the inhibition of PKC-β effectively neutralised the impact of OGD on cytoskeleton in HBMECs, it failed to exert similar effects in OECs (Fig. 3 A-B).

### PKC-β inhibition suppresses oxidative stress in HBMECs while evoking an opposite effect in OECs

**Fig. 4:**
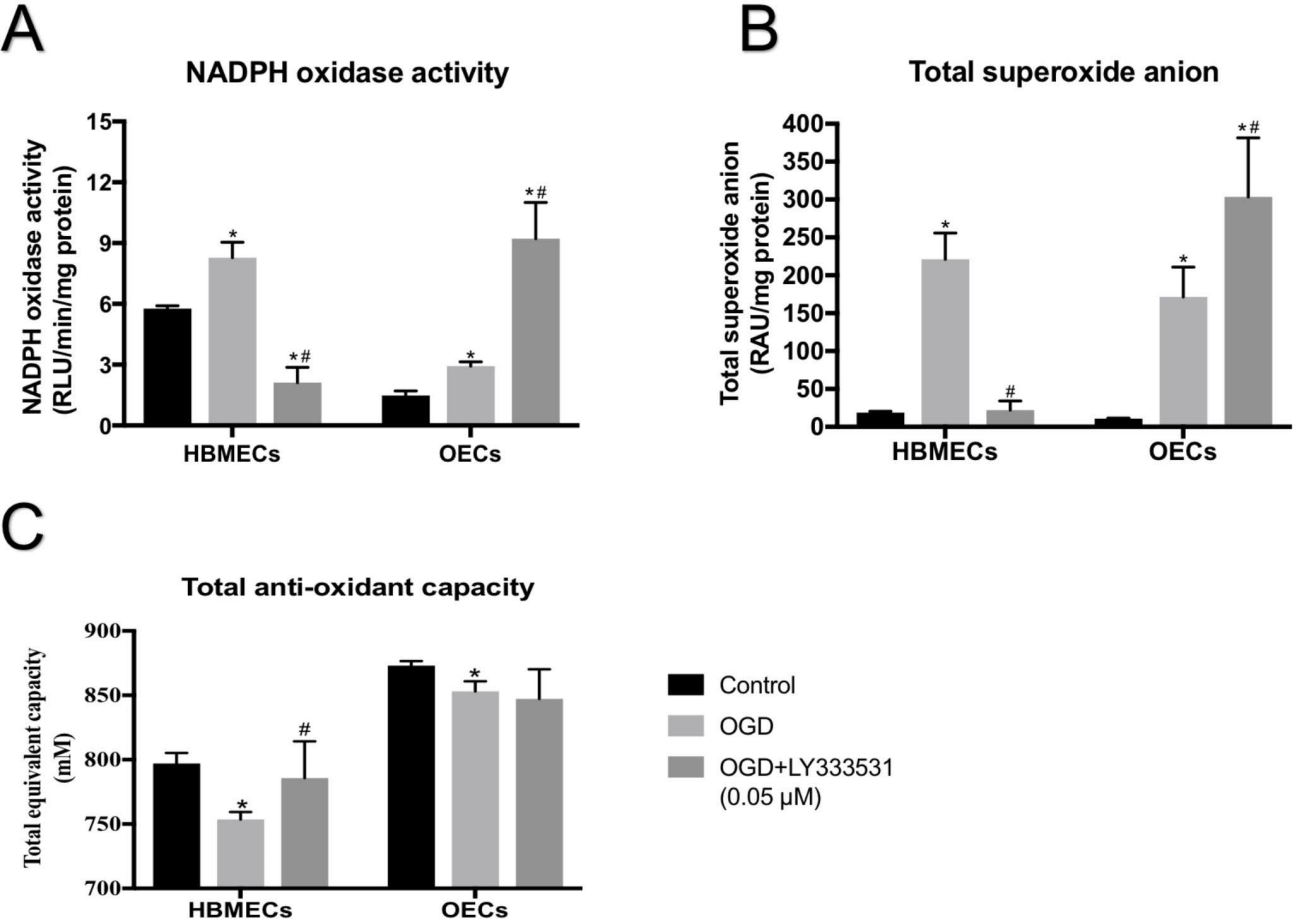
The effect of PKC-β inhibition on NADPH oxidase activity (A), superoxide anion release (B), and total anti-oxidant capacity (C) in HBMEC and OECs subjected to experimental ischaemic injury. While the inhibition of PKC-β using LY333531 (0.05 µM) decreased NADPH oxidase activity and superoxide anion level in HBMEC, it further augmented both parameters in OECs. The presence of LY333531 (0.05 µM) during OGD increased total anti-oxidant capacity in HBMECs but not in OECs. *p < 0.05 versus control, #p < 0.05 versus OGD

Considering that the proper function of BBB mainly depends on homeostasis of redox signalling pathway regulated by pro-oxidant and anti-oxidant enzymes, we further assessed the level of these enzymes in HBMECs or OECs during OGD injury. The basal activity of NADPH oxidase alongside the basal availability of superoxide anion appeared to be significantly lower in OECs compared to those observed in HBMECs. Exposure to OGD markedly increased NADPH oxidase activity and superoxide generation in both cell lines. While inhibition of PKC-β activity by LY333531 significantly diminished both parameters in HBMECs, it accentuated the effect of OGD in OECs (Fig. 4 A-B).

In the context of oxidative stress, the present study also demonstrated that, in physiological settings, OECs possess a substantially greater total anti-oxidant capacity compared to HBMECs. While OGD markedly decreases total anti-oxidant capacity in both cell types, co-treatment of cells with OGD and LY333531 produce cell-type specific outcome i.e. increases in HBMECs and no effect in OECs (Fig. 4 C).

### PKC-β inhibition differentially regulates apoptosis of HBMECs and OECs

**Fig. 5:**
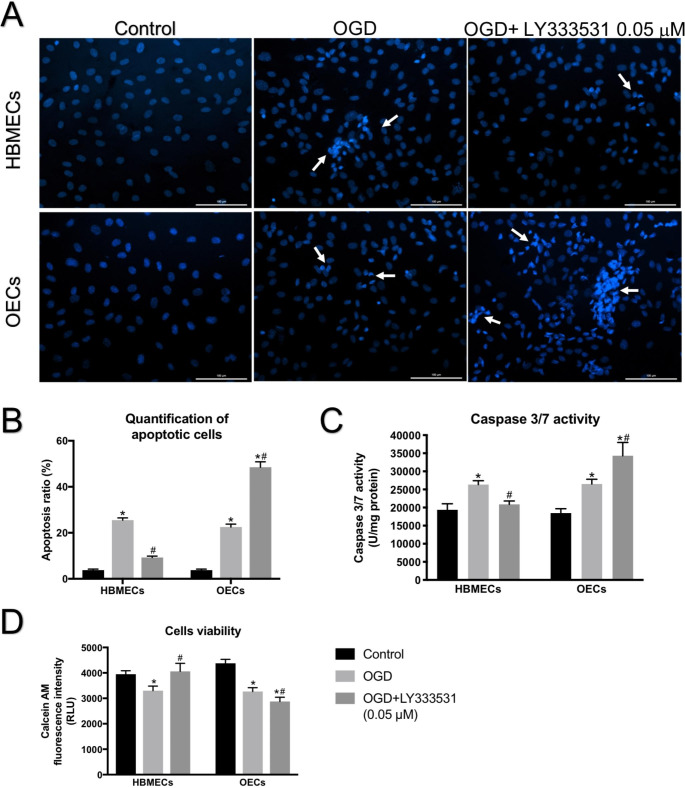
The effects of PKC-β inhibition on cellular apoptosis and viability rates. Representative Hoechst staining showing increases in apoptosis of HBMEC and OECs (indicated by white arrows) exposed to OGD (A). Data graphs showing the quantification of apoptotic cells (B). In HBMECs, treatment with LY333531 (0.05 µM) decreased the impact of OGD on caspase 3/7 activity (C) and thus enhanced their viability (D), it produced the opposite effects in OECs. *p < 0.05 versus control, #p < 0.05 versus OGD. Scale bar: 100 μm

Since physical loss of cells might contribute to the cerebral barrier damage during or after an ischaemic injury, the levels of DNA fragmentation, pro-apoptotic caspase-3/7 enzyme activities, and cell viability were also examined in the current study. OGD significantly increased the number of apoptotic nuclei, determined by Hoechst staining and indicated with white arrows, in both HBMECs and OECs. While co-treatment with LY333531 decreased, but not normalised, the number of HBMECs that underwent OGD-evoked apoptosis, it radically elevated the number of apoptotic OECs (Fig. 5 A-B). The alterations in pro-apoptotic caspase-3/7 enzyme activities in HBMEC and OECs were reflective of those seen in Hoechst staining (Fig. 5 C). In concert with these findings, co-application of LY333531 with OGD enhanced the viability of HBMECs while decreasing that of OECs as evidenced by measurements of Calcein AM fluorescence (Fig. 5D).

### Low dose of LY333531 displays lesser damages

**Fig. 6:**
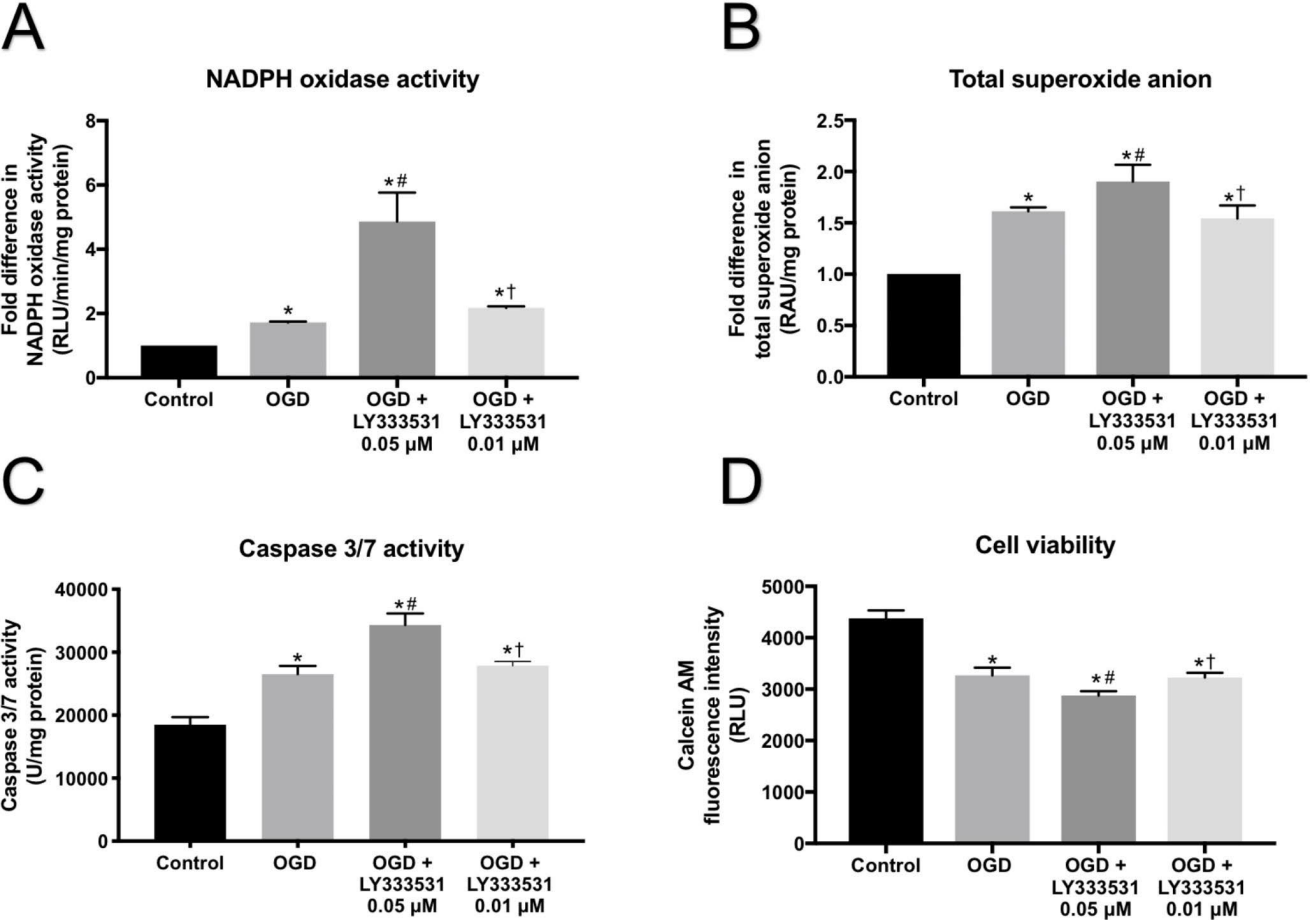
Comparative analyses of the effects of therapeutic and low doses of LY333531 on key elements related oxidative stress and cellular viability in OECs. Exposure to a lower dose of (0.01 µM) LY333531 significantly reduced NADPH oxidase activity (A), superoxide anion production (B), and caspase 3/7 activity (C), and increased rate of survival (D) compared to their counterparts treated with 0.05 µM LY333531. *p < 0.05 versus control, #p < 0.05 versus 0.05 µM dose

Our previous studies have shown that at the specific concentration employed in this study (0.05 µM), LY333531 effectively suppresses total PKC activity and protects the integrity and function of the BBB, composed of HBMECs, HAs and HPs, under laboratory settings of hyperglycaemia and ischaemia-reperfusion injury (Shao and Bayraktutan 2013, 2014; Mathur and Bayraktutan 2016). However, in the current study, this dose produced remarkable increases in both NADPH oxidase activity and superoxide anion generation in OECs subjected to OGD, implying that this particular concentration may abolish the activity of PKC and its downstream targets and therefore trigger a negative feedback mechanism in this particular cell line. In support of this notion, experiments performed with a low dose of LY333531 (0.01 µM) significantly decreased NADPH oxidase activity, superoxide anion release, and caspase 3/7 activity in OECs compared to those treated with high dose LY333531 (Fig. 6 A-C). Similarly, low dose LY333531 produced a substantial increase in cell viability compared to cells treated with high dose LY333531 (Fig. 6D).

## Discussion

Considering the close association between the extent of BBB disruption and severity of stroke and also that cerebral endothelial cells constitute the main cellular element of the BBB, it is of crucial importance to restore endothelial integrity and BBB function after an ischaemic stroke to minimise death and disability (Yu et al. 2015; Kadir et al. 2020). In this context, inhibition of PKC-β by LY333531 (0.05 µM) has been shown to attenuate the deleterious impact of ischaemic injury and hyperglycaemia on endothelial integrity and BBB function (Shao and Bayraktutan 2013; Mathur and Bayraktutan 2016). Since circulatory EPCs continually detect, repair, and replace dead or dying cerebral endothelial cells, the current study has examined whether and how inhibition of PKC-β signalling pathway with LY333531 affects the barrier-forming capacity of OECs, their functional subtype of EPCs, as compared with that of resident endothelial cells in cerebral microvasculature i.e. HBMECs using an experimental model of ischaemic injury. To address this specific question, two different in vitro models of human BBB were established by co-culture of human astrocytes and pericytes with HBMECs or OECs and subjected to oxygen-glucose deprivation (OGD) in the absence or presence of LY333531.

The sudden loss of oxygen and nutrient supply mimicked by OGD injury compromised the integrity and function of both models and decreased transendothelial electrical resistance while increasing the paracellular flux across the barrier. Structural changes enforced upon cytoskeleton by the emergence of stress fibres alongside the dramatic elevations observed in apoptosis and oxidative stress stemming from excessive NADPH oxidase activity and superoxide anion production provide some mechanistic understanding for the ischaemia-mediated failure of both barriers. Contrary to the disruptive effect of OGD on BBB, the impact of OGD and LY333531 co-treatment on BBB was dictated by the type of endothelial cell used to establish them. Namely, while inhibition of PKC-β protected the integrity and function of BBB established with HBMECs, it exacerbated the extent of damage in BBB established with OECs by negating or accentuating the impact of OGD on cytoskeletal remodelling and oxidative stress / apoptosis, respectively. Since, similar to HBMECs, OECs also possess cobblestone morphology and display typical endothelial cell characteristics like ability to take up Dil-Ac-LDL, bind to FITC-UEA-1, form coherent tubular structure on matrigel, and establish equally tight and functional barriers in vitro, these findings were somewhat unexpected.

The differential response between mature endothelial cells and progenitor cells is not unique to ischaemic injury. While the suppression of oxidative stress by agents targeting NF-κB (via PDTC), NADPH oxidase (via DPI or apocynin), and mitochondrial complex I (via rotenone) appeared to exacerbate the suppressive effects of hyperglycaemia on eEPC and OEC viability and proliferation rates (Chen et al. 2007), targeting of the same mechanisms attenuated such detrimental effects in mature endothelial cells, including HBMECs, and enhanced the overall unity and function of BBB (Ulker et al. 2004; Weidig et al. 2004; Allen and Bayraktutan 2009). NADPH oxidase represents one of the key downstream targets of PKC-β and constitutes the main enzymatic source of oxidative stress in vasculature and vascular disease (Bayraktutan 2005b). Similar to our study, knockdown of a specific subunit of NADPH oxidase i.e. Nox2 has also been shown to induce the appearance of thick actin stress fibres in EPCs (Urao et al. 2008). Furthermore, induction of hindlimb ischaemia in mice lacking Nox2 has been associated with a substantial reduction in the number of circulating EPCs as well as with an impaired vascular regeneration (Urao et al. 2008; Schröder et al. 2009). Inhibition of other NADPH oxidase isoforms that have relevance to vascular events, notably that of Nox4, is also implicated in increased rate of cellular death induced by pro-inflammatory cytokine, TNF-α and diminished proliferative and migratory capacity of EPCs (Hakami et al. 2017). Despite suppression of overall NADPH oxidase activity by relatively specific wide-spectrum inhibitors, including VAS2870 or DPI, has largely been linked with diminished hypoxia-mediated EPCs dysfunctions (Liu et al. 2017), reports showing increases in migratory and tube-forming capacity of OECs upon stimulation of PKC signalling pathway by phorbol myristate acetate (PMA, 10 nM) also exist. For instance, administration of PMA-stimulated OECs to mouse model of hindlimb ischemia has been shown to augment neovascularisation by regulating the expression of NF-κB, MMP-2, and MMP-9 (Wu et al. 2019). Other studies have also shown that higher doses of PMA (100 nM) produces about 7-fold increase in the adhesion rate of EPCs to the endothelium and various components of extracellular matrix such as fibronectin, collagen, and vitronectin (Powerski et al. 2011). In contrast to these findings, our previous studies had reported significant increases in barrier damage, oxidative stress, and apoptosis of cerebral endothelial cells in response to higher concentrations of PMA (Shao and Bayraktutan 2014; Rakkar and Bayraktutan 2016). In short, targeting PKC-β pathway or its downstream effectors, most notably NADPH oxidase, may exert distinctive cellular and molecular effects in different cells including mature cerebral endothelial cells and OECs.

In physiological settings, ROS act as signalling molecules and help maintain cellular homeostasis through regulation of cell proliferation, migration, differentiation, and gene expression. Hence, any sharp elevations or decline in NADPH oxidase activity and ensuing superoxide anion release, the foundation molecule of all ROS, can potentially impair endothelial integrity and function (Abid et al. 2000; Zanetti et al. 2001; Bayraktutan 2005a). Accumulating evidence including the findings of the current study show that, compared to mature endothelial cells, OECs possess inherently low basal levels of NADPH oxidase activity and superoxide anion and high level of total anti-oxidant capacity accompanied by increases in activity of antioxidant enzymes manganese superoxide dismutase, glutathione peroxidase, and catalase (Sattler et al. 1999; Dernbach et al. 2004; He et al. 2004). These findings imply that OECs may cope better with the oxidative stress induced by the ischaemia-reperfusion injury and therefore may make effective therapeutics to mitigate post-stroke barrier damage to maintain neurovascular homeostasis. Although suppression of NADPH oxidase by specific targeting of Nox2 or Nox4 subunit has been correlated with significant decreases in ROS level in EPCs (Urao et al. 2008; Schröder et al. 2009; Hakami et al. 2017), the inhibition of PKC-β in the current study led to dramatic increases in NADPH oxidase activity and superoxide anion release. It is possible that the inhibition of PKC-β may completely neutralise the minimal basal oxidant activity and superoxide anion generation in OECs and may, as a consequence, trigger a negative feedback mechanism to compensate these changes in OECs. In support of this notion, treatment with a low dose of LY333531 (0.01 µM) during OGD injury significantly attenuated NADPH oxidase activity, superoxide anion production and caspase-3/7 activities and enhanced OEC viability compared to the therapeutic dose (0.05 µM). However, the levels of NADPH oxidase and caspase-3/7 activities as well as superoxide anion generation remained markedly above those seen in control cells. In light of the well-acknowledged close correlation between the extent of endothelial (barrier) dysfunction and the levels of NADPH oxidase and local availability of superoxide anion, it was safe to assume that disruptions in cytoskeletal organisation and BBB permeability would persist with the lower concentration of LY333531 (Demirci et al. 2008; Rakkar et al. 2014).

In conclusion, this study has demonstrated that OECs share the same characteristics as the mature endothelial cells and can form functional BBB as mature endothelial cells as ascertained by similar readings for TEER and paracellular flux. Secondly, the inhibition of PKC-β attenuated the deleterious impact of OGD injury on the integrity and function of BBB formed by HBMECs by alleviating oxidative stress, cytoskeleton remodelling, and apoptosis but showed the opposite effect on BBB formed by OECs. Thirdly, significantly more damaging effects of LY333531 on pro-oxidant NADPH oxidase, cell viability, and apoptosis at higher and so-called therapeutic concentrations suggest a close scrutiny of PKC-β signalling pathway while using OECs as a cellular restorative strategy.

There are some limitations to the current study. Considering that a lower dose of LY333531 also produced significant reductions in NADPH oxidase activity and superoxide anion level, dose-escalation studies to determine the actual effect of PKC-β inhibition under physiological and pathological condition is worthy of future exploration. Moreover, bearing in mind that OECs’ characteristics are determined by their passage number (high vs. low passage number), source (umbilical cord vs. peripheral blood), and isolation procedure (FACS vs. cell culture) (Eggermann et al. 2003; Medina et al. 2013; Gao et al. 2017), the impact of targeting PKC/oxidative-stress pathway based on these factors may provide better insight into the correlation between targeting this pathway and OECs. In addition, since different ROS influence cellular function differently (Li et al. 1997), quantification of other ROS may be of value in future studies.

## Data Availability

The datasets used and analysed during the current study are available from the corresponding author on reasonable request.
